# A systematic literature review of the key challenges for developing the structure of public health economic models

**DOI:** 10.1007/s00038-015-0775-7

**Published:** 2016-01-08

**Authors:** Hazel Squires, James Chilcott, Ronald Akehurst, Jennifer Burr, Michael P. Kelly

**Affiliations:** School of Health and Related Research, University of Sheffield, Sheffield, UK; BresMed, Sheffield, UK; Primary Care Unit, Institute of Public Health, University of Cambridge, Cambridge, UK

**Keywords:** Public health, Economic model, Methods, Complexity, Model structure, Literature review

## Abstract

**Objectives:**

To identify the key methodological challenges for public health economic modelling and set an agenda for future research.

**Methods:**

An iterative literature search identified papers describing methodological challenges for developing the structure of public health economic models. Additional multidisciplinary literature searches helped expand upon important ideas raised within the review.

**Results:**

Fifteen articles were identified within the formal literature search, highlighting three key challenges: inclusion of non-healthcare costs and outcomes; inclusion of equity; and modelling complex systems and multi-component interventions. Based upon these and multidisciplinary searches about dynamic complexity, the social determinants of health, and models of human behaviour, six areas for future research were specified.

**Conclusions:**

Future research should focus on: the use of systems approaches within health economic modelling; approaches to assist the systematic consideration of the social determinants of health; methods for incorporating models of behaviour and social interactions; consideration of equity; and methodology to help modellers develop valid, credible and transparent public health economic model structures.

**Electronic supplementary material:**

The online version of this article (doi:10.1007/s00038-015-0775-7) contains supplementary material, which is available to authorized users.

## Introduction

An increasing number of model-based assessments of the cost-effectiveness of public health interventions are being commissioned (McDaid and Needle 2006). Modellers trained to develop health economic models of clinical interventions typically apply the same approach for public health interventions, as evidenced by the many published case studies (Weatherly et al. 2009). However, it has been argued that economic modelling within public health raises a number of different methodological challenges compared with economic modelling of clinical interventions (Weatherly et al. 2009).

Weatherly et al. (2009) undertook a review around the challenges of applying standard methods of economic evaluation to public health interventions. The authors identified challenges focusing upon issues of methodological and parameter uncertainty. However, one key concern for modellers is model structuring decisions (Chilcott et al. 2010). Modellers make decisions about what is included and excluded from the model, and how the relationships between inputs and outputs are captured (Stevenson et al. 2012). An inappropriate model structure may lead to poorly informed policy decisions, resulting in inefficient allocation of scarce resources. This paper reviews the key methodological challenges for developing the structure of public health economic models in order to guide future research. We draw upon multidisciplinary literature to develop ideas identified by the formal literature review in more depth.

## Methods

### Formal literature review

The traditional Cochrane search aims to identify all studies that meet pre-specified inclusion and exclusion criteria (Lefebvre et al. 2011). However, methodological reviews often require alternative search strategies which allow the scope of the search to develop as the reviewer’s understanding of the domain increases (Black et al. 1998), with the aim of using the reviewing process to enhance understanding. Thus, in this investigation, papers have been identified using an iterative approach to searching (Paisley 2012). This was not a Cochrane type systematic review; the intention was to identify key relevant studies.

In order to develop an initial understanding of potential methodological issues: (1) papers relating to economic evaluation resulting from the work of the Centre for Public Health Excellence at NICE were identified by searching Medline for the names of authors identified on the NICE website; (2) the publications written by the Public Health Research Consortium, a collaboration between eleven UK institutions to strengthen the evidence base for interventions to improve health, were hand searched; and (3) a search in Medline for terms relating to problems/challenges, public health and economic modelling was undertaken. This was not limited by country. Following this initial search, key public health journals were searched (*Journal of Public Health*, *European Journal of Public Health*, *American Journal of Public Health*, *International Journal of Public Health*) using search terms relating to economic evaluation (see supplementary material for full search terms).

**Fig. 1:**
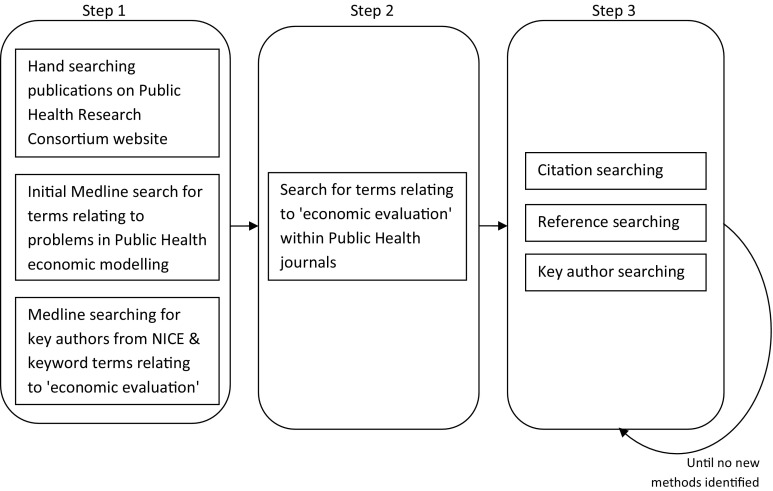
Methods for formal literature search of key challenges in public health economic modelling

All of the retrieved literature was screened at title and abstract level for potential relevance, and the full paper was retrieved where insufficient detail was provided within the abstract. For those considered relevant to the review, citation searching (Scopus), reference searching and key author searching (Medline, Scopus) was undertaken. The search included additional key information presented within “grey literature”, including relevant working papers and presentations from workshops/conferences. The process was repeated iteratively until theoretical saturation. The search was undertaken in December 2010 and citation searching of all of the included papers was repeated in August 2013. Figure 1 shows the methods for the literature search.

Papers were included if they described methodological challenges associated with developing the structure of public health economic models. A relevant paper by Weatherly et al. (2009) was based upon a more extensive report by Drummond (2005) and part of the report presented a systematic review of economic evaluations of public health interventions. The report identified and described the results of three similar systematic reviews; West et al. (2003), Rush et al. (2004) and McDaid and Needle (2006). The main limitations identified by the four systematic reviews were: many different outcome measures are used making comparison difficult; the perspective adopted is often too narrow; and many studies adopt a limited time horizon. The included theoretical papers identified all of these. Consequently, published case studies of economic evaluations within public health and these systematic reviews were not included within this review as it was considered that they were unlikely to offer any new methodological challenges. In addition, papers which considered methodological challenges which are external to model development, such as valuing equity or health outcomes (as against the use of them within a model) were excluded. This is because these are not issues which would change the way models are developed. “Grey literature” was excluded if the content was already published in a peer reviewed journal.

### Multidisciplinary literature search

Because of the iterative nature of the formal literature search, which used the reviewing process to enhance understanding, the reviewers’ awareness of potentially relevant literature was broad in contrast to a focused Cochrane type search. Thus, during the searching and data extraction process the reviewers were aware of extensive research that had been undertaken within other disciplines that the health economics literature only briefly raised. Exploiting this broader literature offers a deeper understanding of each of these issues, which may facilitate the aim of setting an agenda for future research within the health economic context. For each such issue, either the seminal sources within that field were identified or an additional targeted search was undertaken within the relevant discipline.

## Results

### Formal literature review

Fifteen articles identified from the search were considered to be relevant. The articles were divided into three categories, shown in Table 1, which emerged from the reviewing process as being key themes that have each been focused upon by communities of researchers. Many of the articles are opinion pieces or response papers rather than full methodological papers due to this being a relatively new area of research. A summary table of the included articles is shown in the supplementary material.

**Table 1:** Number of relevant articles per key challenge in public health economic modelling identified

Author (year)	Key challenge		
	Inclusion of non-healthcare costs and outcomes	Inclusion of equity	Complex systems and multi-component interventions
Anderson (2010)	✓		✓
Claxton et al. (2007)	✓		
Cookson et al. (2009a)		✓	
Cookson et al. (2009b)		✓	
Kelly et al. (2005)	✓		✓
Mooney (2007)	✓		
Plsek and Greenhalgh (2001)			✓
Richardson (2009)		✓	
Rickles et al. (2007)			✓
Shiell (2007)	✓		✓
Shiell (2009)		✓	
Shiell and Hawe (1996)			✓
Smith and Petticrew (2010)	✓		✓
Weatherly et al. (2009)	✓	✓	✓
Whitehead (2010)			✓
Total per category	7	5	9

#### Inclusion of non-healthcare costs and outcomes (7 articles)

Shiell (2007), Anderson (2010), Smith and Petticrew (2010) and Mooney (2007) argue that the cost-effectiveness of public health interventions can be underestimated if all health and non-health impacts of an intervention are not considered. It may not be appropriate to simply identify these outcomes qualitatively within the report (as recommended by Drummond (2005) with reference to health technology assessments) due to the substantial impact non-health effects could have upon model results within public health. Shiell (2007), however, recognises that many costs and benefits cannot be, or are difficult to measure within public health. Smith and Petticrew suggest that public health economic modelling should focus upon broader outcomes such as ‘happiness’ as one way of attempting to capture these broader costs and outcomes. Kelly et al. and Weatherly et al. also suggest that the QALY outcome measure may be insufficient for economic evaluations of public health interventions because it does not capture the mental and social outcomes associated with some public health interventions or non-health outcomes such as education or crime. Both papers suggest as a potential solution a cost-consequence analysis from the perspective of each sector as a supplementary analysis. This is also recommended by Anderson. However, there remain practical issues relating to the way in which decision-makers should use this information to compare interventions, which are not addressed within these papers. Kelly et al. also suggest that discrete choice experiments could be used to provide a broader outcome measure than the QALY.

**Fig. 2:**
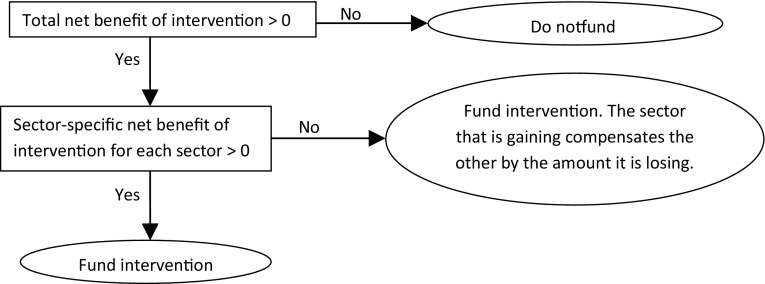
Compensation test approach

Claxton et al. (2007) propose an alternative solution for the inclusion of intersectoral costs and benefits. This involves estimating the net benefit of the public health interventions from all relevant sectoral perspectives and applying a compensation test as shown in Fig. 2. Whilst this approach seems theoretically reasonable, the paper does not try to address practical issues; valuation methods, that metrics and thresholds differ by sector and the cooperation of other sectors would be required for this approach to be feasible.

An opinion piece by Mooney (2007) suggests that it may be difficult for stakeholders to agree upon the benefits and risks associated with a public health intervention and as a result all relevant outcomes may not be included within economic evaluations. For example, is a health promotion campaign successful if people are more informed but do not change their lifestyle? The author argues that the ‘costs’ of necessary changes in lifestyle need to be considered (e.g. the ‘cost’ of getting up at 6 a.m. to go to the gym). Methods for determining relevant costs and benefits are not suggested.

These papers all highlight a number of difficulties in defining relevant costs and outcomes for the evaluation. Only three of the seven studies suggest potential methods for dealing with this and all three involve presenting the model results in an alternative format, rather than how these costs and outcomes might be identified (Claxton et al. 2007; Kelly et al. 2005; Weatherly et al. 2009). This is despite all authors highlighting that one of their key concerns is in identifying and including all relevant costs and outcomes. The presentation of alternative results is helpful only if relevant costs and outcomes have been incorporated within the analysis.

#### Inclusion of equity (5 articles)

The UK Government aims to increase overall health and reduce health inequities (Department of Health 2010). Kelly et al. (2005) argue that these two objectives may require different interventions; thus there is a greater need to develop methods for including equity considerations within economic evaluations of public health interventions. Cookson et al. (2009a) discuss the need for the explicit incorporation of equity within economic evaluation of public health interventions and propose four potential methods for doing this. This is followed by a series of responses by Richardson (2009), Shiell (2009), and the original authors (Cookson et al. 2009b). The authors highlight how health inequity reduction is a key policy objective in the field of public health, yet equity considerations are not typically addressed within economic evaluations. The four methods for considering equity within economic evaluations of public health interventions proposed by Cookson et al. are:

1. Identification of relevant equity considerations and a review of existing literature to provide qualitative discussion on equity issues;
2. Quantitative analysis of key subgroup data from trials, where available, around the impact of the intervention upon health inequities;
3. Estimating the opportunity cost of including equity considerations in terms of health foregone (i.e. the comparison of health foregone if adopting the equitable option with that of maximising health);
4. Valuing health inequality reduction by quantitatively weighting health outcomes according to equity considerations.

The authors conclude that it is not presently possible to specify the most appropriate approach and that testing of each is required. Richardson (2009) highlights that the analysis of equity within economic evaluation is underdeveloped given that Cookson et al. have proposed approaches 1 and 2 above.

Shiell (2009) argued that health inequality reduction is highly unlikely if interventions are confined to individually based clinical and lifestyle interventions (i.e. ‘downstream’ interventions). Trying to value them might not therefore be that helpful. However, undertaking primary research and modelling of interventions tackling the social determinants of health (i.e. ‘upstream’ interventions) which have much greater potential to achieve a reduction in health inequalities, would be worthwhile. Upstream interventions are those which affect the whole population like minimum unit pricing of alcohol, lowering the salt content in processed foods and non-health sector interventions like providing affordable housing.

All of these papers highlight the importance of considering equity in some capacity within economic evaluations of public health interventions; however, there is currently no agreement over the most appropriate approach.

#### Modelling complex systems and multi-component interventions (9 articles)

Shiell et al. (2008) define a complex system and distinguish this from complex interventions. They state a complex system ‘is adaptive to changes in its local environment, is composed of other complex systems and behaves in a non-linear fashion’, for example the stock market. They define a complex, or multi-component, intervention as ‘built up from a number of components, which may act both independently and inter-dependently’ as defined by the Medical Research Council (Craig et al. 2008). Shiell et al. argue that whilst multi-component interventions are more difficult to evaluate, methodologies for economic evaluation of multi-component interventions are not fundamentally different since it is not necessary to understand how the intervention works within an economic evaluation. However, Kelly et al. (2005) suggest that from a policy perspective it is important for a model to address what aspects of an intervention make it successful or unsuccessful. This is to help decision-makers understand how different approaches may be used to overcome barriers to change, whether interventions may be generalisable in other settings and where the impact on specific subgroups needs to be modelled.

Shiell et al. (2008) argue that the evaluation of interventions within complex systems presents new methodological challenges, stating that the usual approach is to assume that the effects of an intervention can be assessed without considering the impact of the environment upon its effectiveness (i.e. social structure and people’s interactions). Similarly, Plsek and Greenhalgh (2001) discuss the challenge of complexity in healthcare systems and suggest that the science of complex adaptive systems (also termed ‘dynamically complex systems’) is appropriate for addressing this challenge. This means modelling a system by considering the behaviour of the parts and the relationships between those parts (Miller and Page 2007). Whilst the theory within both of these papers is logical, they do not go further to describe how the science of complex adaptive systems could be used or to test this theory.

Similarly, Anderson (2010) suggests that some of the key reasons for public health economic evaluation being more challenging than modelling clinical interventions are due to the interventions being multi-component, with tailored, dynamic and evolving implementation which may be at the community/population level rather than the individual level. He makes the point that, within public health, there are long causal chains and the causal mechanisms may be social and behavioural as well as biological, making results of models of the ‘average’ person potentially meaningless. Thus models of human behaviour will be useful in developing the economics.

Shiell and Hawe (1996) argue that for interventions which have the community rather than the individual as the focus, there may be additional community impacts, distinct from the aggregate outcomes of individuals, which need to be captured. If these broader community impacts, such as empowerment are excluded from the model, the cost-effectiveness of these interventions will be systematically underestimated. Similarly, Smith and Petticrew (2010) argue that there is a need to focus on the effects of the interventions upon communities and populations, as well as on individual effects. However, Whitehead (2010) argues that public health evaluations have been undertaken using a macro-level analysis, such as within tobacco control, and that it is the funders of public health economic modelling who encourage a micro-level approach rather than the analysts. Again, no potential solutions are provided.

Rickles et al. (2007) consider how causality is established within complex intervention research such as public health. They explain that outcomes will be affected by manipulation of variables only where causation, rather than correlation, is present. Within public health, outcomes are not only dependent upon characteristics of the individual, but also upon social structure. The authors suggest that effectiveness is difficult to estimate even with a randomised controlled trial because of the problem with identifying and controlling for all relevant variables. They argue that simulation studies do not provide a better solution since it is necessary to assume a causal structure and it is not possible to know the unknown variables in the system. Whilst these are all relevant issues, decision-makers need to make policy decisions in the face of these uncertainties (Stevenson et al. 2012). A model makes these assumptions explicit so that they can be discussed and debated and allows exploration of the implications of alternative assumptions (Pidd 2009). In addition, being explicit about what is ‘known’ within a model provides a good starting point for understanding what is not known. Thus, whilst establishing causality may present challenges, rather than dismissing the use of models, it may be useful to consider the implications of these challenges for model development and validation. Weatherly et al. (2009) and Kelly et al. (2005) argue for more use of econometric methodology for analysing non-experimental data including techniques such as time series analysis, propensity score matching and difference-in-difference techniques.

Within a workshop presentation, Anderson (2010) suggests that there are two widely divergent approaches being employed for public health modelling; “back of a fag packet” (i.e. very simple models) or “cerebral meltdown” [for example, the Foresight obesity system map (2007)]. Algorithmic methods exist to identify key factors from a large number of causally related factors which could be employed to limit the scope of the model, although these are not generally employed within health economics (Squires 2014). Anderson highlights that the population of interest, the starting point for the simulation and the care pathway may be less well defined in public health and evidence is usually short-term and inconsistent between studies of the interventions. He therefore suggests that modelling should potentially be more exploratory, with results presented in terms of sensitivity analyses rather than a ‘base case’. He also indicates that cohort decision trees and Markov models, which are typically employed within Health Technology Assessment, may not be adequate due to the dynamic complexity of public health systems. Decision trees and Markov models are less flexible for modelling more complex systems compared with other techniques such as system dynamics (SD), discrete event simulation (DES), or agent based modelling (ABM). SD can be used to more easily describe dynamic complexity at a population level, whilst DES and ABM describe the interactions of heterogeneous individuals with their environment, with ABM more easily enabling spatial aspects and interaction between individuals to be modelled. There is therefore a need to justify the model structures which are developed and the level of complexity employed.

All of these papers highlight the complexity associated with the assessment of public health interventions and that current approaches to health economic modelling are not sufficient to deal with this. It is proposed that the science of complex adaptive systems, or dynamic complexity, may be helpful; however, the argument is underdeveloped. Methods for working with and analysing dynamically complex systems should be explored. Issues around describing causal relationships are raised and it is suggested that more use should be made of econometric methodology. Since the causal mechanisms may be social and behavioural as well as biological, models of human behaviour may be useful. It is suggested that more complex types of models may be required and that the analysis may need to be more exploratory. Finally, the model structure chosen should be justified.

### Multidisciplinary literature review

Substantial research has been published within other non-economic disciplines in relation to the three issues raised within this review, including dynamic complexity (Systems Thinking literature), the social determinants of health (public health literature) and models of human behaviour (psychology and sociology literature). This literature was explored to understand the current status of this research and to assess the research implications for public health economic modelling.

#### Dynamic complexity

**Fig. 3:**
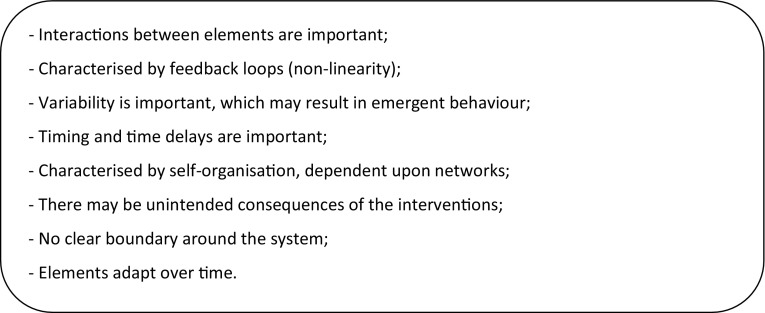
Characteristics of a dynamically complex system

A key message from the review is the methodological challenge related to public health systems being complex. Based upon seminal books about complex adaptive systems, otherwise termed dynamic complexity, and systems thinking by Miller and Page (2007) and Sterman (2000), key characteristics of a dynamically complex system are shown in Fig. 3. As described within the review, current approaches for public health economic modelling do not tend to address all of these. There is a need for methods for describing and analysing the complexity of the system in order to capture relevant aspects viably within a model.

A systems approach takes a holistic way of thinking about complex systems, and focuses upon the interactions amongst entities and between entities and their environment (Sterman 2000). It is recognised that by considering one aspect of a system in isolation, there may be unintended consequences which may make the problem worse. A systems approach is likely to be appropriate for modelling these dynamically complex public health systems; however, this is not yet standard practice for evaluating complex interventions (Green 2006; Homer and Hirsch 2006; Joffe and Mindell 2006; Leischow and Milstein 2006; Midgley 2006; Sterman 2006; Trochim et al. 2006). There may be practical issues associated with adopting these systems approaches within public health modelling and further research is recommended.

#### The social determinants of health and health inequities

Many of the key challenges raised within the review relate to the social determinants of health and health inequities. Currently, the health economic literature gives little recognition to these relationships.

Social structure is the result of individual actions (human agency) which create patterns of behaviour (Kelly and Doohan 2012). However, sociologists argue that people’s behaviour generates social structure and at the same time the social structure constrains and enables behaviour (Giddens 1979). At the social level, health is affected by the influences of social patterning, whilst at the individual level, human behaviour and biology are causally linked to disease. It would therefore be insufficient for public health interventions to aim to modify individual behaviour, without consideration of social structure, or to ignore the interaction between the individual and social level when assessing the effectiveness and cost-effectiveness of interventions. The determinants of health have been classified in many different ways, but they tend to include individual, community and population-level influence upon health (Kelly and Doohan 2012).

A recent classification by Kelly and Doohan (2012) includes a brief summary of key existing models of the determinants of health inequities. From these studies, we identified the following key implications for modelling public health interventions:

- Causal relationships should be considered across the individual, community and population determinants of health. This is a difficult task, requiring further theoretical and empirical research. As Kelly, Kelly and Russo (2014) noted, it is critical to capture both the individual and social level. Both public health literature and practice have until recently failed to make this analytic distinction and this seriously impacts on practice and policy effectiveness. We suggest here that it would be extremely useful for modellers to develop an understanding of the broader determinants of health so that they can recognise which determinants and causal relationships are likely to be of key importance for inclusion within the model. Existing approaches to help capture causal relationships between the determinants of health are causal mapping (Joffe and Mindell 2006) and econometric techniques (Weatherly et al. 2009; Kelly et al. 2005).
- The most effective outcomes are likely to result from interventions targeted simultaneously at individual, community and population levels. Whilst this has not generally been the approach taken, there are examples of very successful overarching approaches within public health. For example, tobacco control interventions have included altering laws for selling tobacco and banning smoking in public places, as well as cessation interventions for individual smokers. Similarly, a wide range of interventions have been employed for HIV prevention. However, there are many areas where this multilevel approach has not been taken; most interventions relating to physical activity and diet for example. It is possible within a model to synthesise evidence to compare interventions across all levels, where interactions between interventions can be explored by testing different assumptions. This is illustrated within a recent economic evaluation of diabetes prevention interventions which involved assessing interventions at each of these levels (including soft drinks tax, fruit and vegetable retail provision, workplace dietary interventions, community education programmes, and diet and exercise interventions for high risk individuals) both individually and simultaneously (Breeze et al. 2015).
- The context within which interventions are provided and the stage within the individual life course will impact upon effectiveness. This could be tested within exploratory analysis within a model.
- The modeller should be aware that the determinants may impact upon overall health and health inequities in different ways.

A description of the determinants of health may help to facilitate consideration of non-healthcare costs and outcomes. It could also help identify potential types of interventions, for example those which might impact upon individual health through making community and population-level changes, such as food production, as well as those which might impact upon health through changing individual lifestyle factors. Similarly, subpopulations that might benefit from the intervention could be identified.

#### Models of human behaviour

Within the review it was recognised that describing individual and societal behaviour is important for evaluating public health interventions, yet no studies were identified which considered how human behaviour might be incorporated into health economic models. Literature from the fields of Sociology and Psychology are explored.

Within psychology, hundreds of models of human behaviour have been developed which provide an understanding of the individual factors required for the adoption of a specified behaviour. However, only a small number of these have had empirical applications. A review by Taylor et al. (2006) identified the Health Belief Model, the Theory of Reasoned Action, the Theory of Planned Behaviour and the Trans-Theoretical Model as the most commonly used cognitive models within health promotion. This review suggested that none of these four models adequately capture social, economic or environmental factors as predictors and determinants of health behaviour. Recently, there have been attempts to incorporate human behaviour into mathematical models of public health (Brailsford and Schmidt 2003; Hu and Puddy 2011; Kruger et al. 2013). Within these case studies, the Health Belief Model, the Theory of Planned Behaviour and a questionnaire were used, and there were difficulties with parameterisation in each case. Methodological research around parameterisation and practical implementation is required to employ these behavioural models within public health economic modelling. Currently health economic modelling has largely overlooked the incorporation of psychology models and this could be an important area of further research. Similarly, research about the potential benefits of employing behavioural economics, which integrates psychology with neo-classical economics, may be useful (Thaler and Mullainathan 2008).

Sociology seeks to provide insights into the many forms of relationship between people (including cultural, economic and political) to understand how society works (The British Sociological Association 2013). It provides an evidence-based perspective of society, questioning conventional assumptions within society, and could provide tools for modelling the impact of interactions within society upon outcomes. Within the last decade sociology has been linked with complex adaptive systems to form a discipline defined as sociology and complexity science (SACS) (Castellani and Hafferty 2009). Two of the biggest areas of work within SACS are computational sociology and complex social network analysis. Computational sociology is the use of computationally intensive methods to analyse social systems. To date many of these models have made assumptions about behaviour based upon limited or no data (Hu and Puddy 2011). Complex social network analysis involves the use of a range of techniques including agent-based modelling (individual-level simulation which is made up of agents following a set of rules about their interactions with other agents and their environment) and social network analysis (mapping social networks to understand who is at the hub of the network) (Siebers et al. 2010). These methods have been used to describe the spread of public health problems such as obesity, smoking, alcohol, influenza and HIV (Christakis and Fowler 2007, 2008; Kumar et al. 2013; Rosenquist et al. 2010; Tully et al. 2013). However, they have not commonly been employed within the health economics community. Further development and application of these methods to public health economic evaluation should be explored within future research.

## Discussion

Methodological papers about public health economic modelling have generally only been published since the turn of the twenty-first century and there is debate about the best way to address the challenges as demonstrated by the many opinion pieces and response papers published. Economic evaluations within public health are generally different to economic evaluations of clinical interventions. This is because they usually require the development of models of multi-component interventions with complex causal chains operating within dynamically complex systems, dependent upon the determinants of health; as against models of simple interventions operating within relatively clear system boundaries which generally do not depend upon human behaviour. It is also often much less clear what a ‘good’ outcome of a public health intervention is. In addition, a key objective of public health is to reduce health inequities. Very few of the studies propose any methodology for dealing with the issues they raise, and those that do generally focus upon alternative ways of presenting the model results. Anderson (2010) suggests there is a dichotomy, with some analysts developing very simple public health models and others developing highly complex ones. These very different model structures are generally developed with limited justification for the level of complexity and this is an important gap in current practice highlighted by the review.

Inclusion for this review was not limited by country and thus the results should be internationally relevant. It is possible that the use of additional databases for the searches could have led to identification of more literature; however, the iterative nature of the search should have led to the identification of any relevant studies. The literature considered during the further analysis is multidisciplinary which avoids a parochial perspective about challenges and areas for further research.

Key areas identified for future methodological research are:

- The use of systems approaches for dealing with dynamic complexity and for including non-health costs and outcomes within health economic models;
- An approach for encouraging modellers to be aware of and consider inclusion of the social determinants of health;
- The potential to incorporate models of behaviour from psychology, sociology or behavioural economics within health economic models;
- The development of modelling methods to enable social interactions to be incorporated, such as agent-based simulation and social network analysis;
- Incorporation of equity within the modelling process;
- A methodology to help modellers develop valid, credible and transparent public health economic model structures.

This agenda for future research can inform methods development for public health economic modelling and, in turn, help decision-makers to make appropriate public health policy decisions.

## Electronic supplementary material

Below is the link to the electronic supplementary material.

Supplementary material 1 (DOCX 35 kb)

## References

[CR1] Anderson R (2010) Modelling and evidence synthesis: challenges, value and issues for discussion. Workshop in methods of economic evaluation in public health research. Public Health Research Consortium. Report of the workshop available at http://phrc.lshtm.ac.uk/papers/PHRC_Annual_Report_2009-10.pdf

[CR2] Black N, Brazier J, Fitzpatrick R, Reeves B (1998). Methods for Health Services Research: a state of the art guide.

[CR3] Brailsford S, Schmidt B (2003). Towards incorporating human behaviour in models of health care systems: an approach using discrete event simulation. Eur J Oper Res.

[CR4] Breeze P, Thomas C, Squires H, Brennan A, Greaves C, Diggle P et al (2015) Prevention model: detailed description of model background, methods, assumptions and parameters. HEDS Discussion Paper. https://www.shef.ac.uk/scharr/sections/heds/discussion

[CR5] Castellani B, Hafferty F (2009). Sociology and complexity science: a new field of enquiry.

[CR6] Chilcott J, Tappenden P, Rawdin A, Johnson M, Kaltenthaler E, Paisley S, Papaioannou D, Shippam A (2010) Avoiding and identifying errors in health technology assessment models, 14 edn 10.3310/hta14250 20501062

[CR7] Christakis NA, Fowler JH (2007). The spread of obesity in a large social network over 32 years. N Engl J Med.

[CR8] Christakis NA, Fowler JH (2008). The collective dynamics of smoking in a large social network. N Engl J Med.

[CR9] Claxton K, Sculpher M, Culyer A (2007) Mark versus Luke? Appropriate methods for the Evaluation of Public Health Interventions. Working papers from Centre for Health Economics, University of York

[CR10] Cookson R, Drummond M, Weatherly H (2009). Explicit incorporation of equity considerations into economic evaluation of public health interventions. Health Econ Policy Law.

[CR11] Cookson R, Drummond M, Weatherly H (2009). Explicit incorporation of equity considerations into economic evaluation of public health interventions—reply to Richardson and Shiell. Health Econ Policy Law.

[CR12] Craig P, Dieppe P, Macintyre S, Michie S, Nazareth I, Petticrew M (2008) Developing and evaluating complex interventions: new guidance

[CR13] Department of Health (2010) Healthy lives, healthy people: our strategy for public health in England. https://www.gov.uk/government/uploads/system/uploads/attachment_data/file/216096/dh_127424.pdf. Accessed Jan 2011

[CR14] Drummond MF (2005). Methods for the economic evaluation of healthcare programmes.

[CR15] Foresight (2007) Tackling obesities: future choices, 2nd edn. Government Office for Science

[CR16] Giddens A (1979). Central problems in social theory: action, structure and contradiction in social analysis.

[CR17] Green LW (2006). Public health asks of systems science: to advance our evidence-based practice, can you help us get more practice-based evidence?. Am J Public Health.

[CR18] Homer JB, Hirsch GB (2006). System dynamics modeling for public health: background and opportunities. Am J Public Health.

[CR19] Hu X, Puddy R (2011) Cognitive modeling for agent-based simulation of child maltreatment. Social computing, behavioral-cultural modeling and prediction. Springer, Berlin, Heidelberg, pp 138–146

[CR20] Joffe M, Mindell J (2006). Complex causal process diagrams for analyzing the health impacts of policy interventions. Am J Public Health.

[CR21] Kelly MP, Doohan E, Merson MH, Black RE, Mills AJ (2012). The Social determinants of health. Global health: diseases, programs, systems and policies.

[CR22] Kelly MP, McDaid D, Ludbrook A, Powell J (2005) Economic appraisal of public health interventions. Health Development Agency, London

[CR23] Kelly MP, Kelly R, Russo F (2014). The integration of social, behavioural and biological mechanisms in models of pathogenesis. Perspect Biol Med.

[CR24] Kruger J, Brennan A, Thokala P, Basarir H, Cooke D, Clark M, Bond R, Heller S (2013) Modelling the potential cost-effectiveness of a targeted follow-up intervention to improve glycaemic response following structured training in flexible intensive insulin therapy. DP 13/10 edn

[CR25] Kumar S, Grefenstette JJ, Galloway D, Albert SM, Burke DS (2013). Policies to reduce influenza in the workplace: impact assessments using an agent-based model. Am J Public Health.

[CR26] Lefebvre C, Manheimer E, Glanville J (2011) Chapter 6: searching for studies. In: Higgins JPT, Green S (eds) Cochrane handbook for systematic reviews of interventions version 5.1.0 (updated March 2011). The Cochrane Collaboration, 2009

[CR27] Leischow SJ, Milstein B (2006). Systems thinking and modeling for public health practice. Am J Public Health.

[CR28] McDaid D, Needle J (2006) Economic evaluation and public health: mapping the literature. Cardiff, October 2006. Report prepared for the Welsh Assembly Government Health Promotion Division. Wanless Health Economics Research Programme

[CR29] Midgley G (2006). Systemic intervention for public health. Am J Public Health.

[CR30] Miller JH, Page SE (2007). Complex adaptive systems: an introduction to computational models of social life.

[CR31] Mooney G (2007). Economic evaluation of prevention: we need to do better but first we need to sort out what the good is. Int J Public Health.

[CR32] Paisley S (2012) Identifying evidence to inform decision-analytic models of cost-effectiveness: a qualitative study of information seeking processes and behaviour. PhD Thesis, University of Sheffield

[CR56] Pidd M (2009) Tools for thinking; modelling in management science. John Wiley and Son Ltd, Chichester

[CR33] Plsek PE, Greenhalgh T (2001). Complexity science: the challenge of complexity in health care. BMJ.

[CR34] Richardson J (2009). Is the incorporation of equity considerations into economic evaluation really so simple? A comment on Cookson, Drummond and Weatherly. Health Econ Policy Law.

[CR35] Rickles D, Hawe P, Shiell A (2007). A simple guide to chaos and complexity. J Epidemiol Community Health.

[CR36] Rosenquist JN, Murabito J, Fowler JH, Christakis NA (2010). The spread of alcohol consumption behavior in a large social network. Ann Intern Med.

[CR37] Rush B, Shiell A, Hawe P (2004). A census of economic evaluations in health promotion. Health Educ Res.

[CR38] Shiell A (2007). In search of social value. Int J Public Health.

[CR39] Shiell A (2009). Still waiting for the great leap forward. Health Econ Policy Law.

[CR40] Shiell A, Hawe P (1996). Health promotion community development and the tyranny of individualism. Health Econ.

[CR41] Shiell A, Hawe P, Gold L (2008). Complex interventions or complex systems? Implications for health economic evaluation. BMJ.

[CR42] Siebers PO, Macal CM, Garnett J, Buxton D, Pidd M (2010). Discrete-event simulation is dead, long live agent-based simulation!. J Simul.

[CR43] Smith RD, Petticrew M (2010). Public health evaluation in the twenty-first century: time to see the wood as well as the trees. J Public Health.

[CR44] Squires H (2014) A methodological framework for developing the structure of Public Health economic models. PhD Thesis, University of Sheffield. http://etheses.whiterose.ac.uk/5316/. Accessed May 2015

[CR45] Sterman JD (2000). Business dynamics: systems thinking and modeling for a complex world.

[CR46] Sterman JD (2006). Learning from evidence in a complex world. Am J Public Health.

[CR47] Stevenson M, Tappenden P, Squires H (2012) A position paper on the evaluation of uncertainty in the field of health economic evaluation. Int J Syst Sci, pp 1–9

[CR48] Taylor D, Bury M, Campling N, Carter S, Garfield S, Newbould J, Rennie T (2006) A review of the use of the Health Belief Model (HBM), the Theory of Reasoned Action (TRA), the Theory of Planned Behaviour (TPB) and the Trans-Theoretical Model (TTM) to study and predict health related behaviour change. Report to NICE, 2006. http://www.nice.org.uk/nicemedia/live/11868/44524/44524.pdf. Accessed June 2011

[CR49] Thaler RH, Mullainathan S (2008) Behavioral economics, 2nd edn. The Concise Encyclopedia of Economics. Liberty Fund

[CR50] The British Sociological Association (2013) What is sociology? http://www.britsoc.co.uk/WhatIsSociology/SocHist.aspx

[CR51] Trochim WM, Cabrera DA, Milstein B, Gallagher RS, Leischow SJ (2006). Practical challenges of systems thinking and modeling in public health. Am J Public Health.

[CR52] Tully S, Cojocaru M, Bauch CT (2013). Coevolution of risk perception, sexual behaviour, and HIV transmission in an agent-based model. J Theor Biol.

[CR53] Weatherly H, Drummond M, Claxton K, Cookson R, Ferguson B, Godfrey C, Rice N, Sculpher M, Sowden A (2009). Methods for assessing the cost-effectiveness of public health interventions: key challenges and recommendations. Health Policy.

[CR54] West P, Sanderson D, Redmond S, Taylor M, Duffy S (2003) A critique of the application of cost-effectiveness analysis to public health. Report to inform the Wanless team at HM Treasury (Accessed through personal communication with the authors)

[CR55] Whitehead M (2010). The right wood, but barking up the wrong tree. J Public Health.

