# Supplementary material for: A systematic literature review of the key challenges for developing the structure of public health economic models
Source: Int J Public Health. 2016 Jan 8;61:289–98. doi: 10.1007/s00038-015-0775-7 (PMC4879162; doi:10.1007/s00038-015-0775-7)
Supplement: Supplementary file 1 — Supplementary material 1 (DOCX 35 kb) [file 38_2015_775_MOESM1_ESM.docx]

## Supplementary material for article titled ‘A systematic literature review of the key challenges for developing the structure of Public Health economic models’, International Journal of Public Health; Squires H, Chilcott J, Akehurst R, Burr J, Kelly MP. Corresponding author: [h.squires@sheffield.ac.uk](mailto:h.squires@sheffield.ac.uk), ScHARR, University of Sheffield.

## Part A: Search strategy for systematic literature review of key challenges in Public Health economic modelling

**Stage 1:**

**MEDLINE (1999 – 2010)**

1. "public health".mp
2. challenge$.mp
3. issue$.mp
4. problem$.mp
5. method$.mp
6. "cost-effective$".ti
7. "economic evaluation$".ti
8. 2 or 3 or 4 or 5
9. 6 or 7
10. 1 and 8 and 9

**MEDLINE (1999 – 2010)**

1. Kelly.m.au
2. "cost-effective$".ti
3. "economic evaluation$".ti
4. 2 or 3
5. 1 and 4

**Stage 2:**

**International Journal of Public Health, Journal of Public Health, European Journal of Public Health, American Journal of Public Health (1999 – 2010)**

1. "cost-effective$".mp
2. "economic evaluation$".mp
3. 1 or 2

**Stage 3:**

**Author searching in MEDLINE (1999 – 2010):**

1. Author name
2. "cost-effective$".ti
3. "economic evaluation$".ti
4. 2 or 3
5. 1 and 4

## Part B: Summary of included papers

| **Inclusion of non-healthcare costs and outcomes** | | | |
| --- | --- | --- | --- |
| **Author (year)** | **Title (type of article)** | **Key issues raised** | **Recommended approach** |
| Claxton *et al.* (2007)^60^ | Mark versus Luke? Appropriate methods for the Evaluation of Public Health interventions. (Working paper) | Costs and benefits across sectors should be incorporated. | Compensation test approach. |
| Kelly *et al.* (2005)^59^ | Economic appraisal of public health interventions. (Briefing paper) | -QALY outcome may not be a sufficient measure;  -Greater need to develop methods for including equity considerations;  -It is important for a model to address what aspects of an intervention are successful/ unsuccessful. | -Cost consequence analysis from perspective of each sector as additional analysis; Discrete choice experiments may be used for valuation.  -None provided;  -None provided. |
| Mooney (2007)^61^ | Economic evaluation of prevention: we need to do better but first we need to sort out what the good is. (Opinion piece) | Relevant costs and benefits may be difficult to agree upon. | None provided. |
| Shiell (2007)^62^ | In search of social value. (Opinion piece) | Insufficient to qualitatively include non-health impacts. | None provided. |
| Smith and Petticrew (2010)^63^ | Public health evaluation in the twenty-first century: time to see the wood as well as the trees. (Full journal article) | -Public Health economic modelling should focus upon broader outcomes such as ‘happiness’;  -There is a need to focus on the direct and indirect effects of the interventions upon communities and populations, as well as on individual effects. | -None provided;  -None provided. |
| Weatherly *et al.* (2009)^58^ | Methods for assessing the cost-effectiveness of public health interventions: Key challenges and recommendations. (Full journal article) | Four key methodological challenges:  -Quantifying the effectiveness of interventions;  -Measuring and valuing outcomes;  -Inclusion of intersectoral costs and consequences;    -Inclusion of equity. | -More use could be made of techniques for analysing non-experimental data (eg. econometric analysis);  -Compensation test approach (Claxton *et al*.)  -Cost consequence analysis from perspective of each sector as additional analysis;  -None provided. |

| **Inclusion of equity** | | | |
| --- | --- | --- | --- |
| **Author (year)** | **Title (type of article)** | **Key issues raised** | **Recommended approach** |
| Cookson *et al.* (2009a)^64^ | Explicit incorporation of equity considerations into economic evaluation of public health interventions. (Full journal article) | -There is a need for explicit incorporation of equity;  -Policy makers would not fund cost-effective interventions if they infringe individual liberties or discriminate against the individual;  -Society would be willing to pay more per QALY gained for certain groups such as children, the severely ill and the socioeconomically disadvantaged. | 4 proposed methods: (1) Qualitative discussion around relevant equity issues; (2) Quantitative evidence around the impact of the intervention upon health inequalities; (3) Estimating the opportunity cost of equity considerations in terms of health outcomes willing to forego; (4) Equity weighting of health outcomes. |
| Cookson *et al.* (2009b)^67^ | Explicit incorporation of quity considerations into economic evaluation of public health interventions – Reply to Richardson & Shiell (Response article) | Response to above issues. | Four proposed methods above. |
| Richardson (2009)^65^ | Is the incorporation of equity considerations into economic evaluation really so simple? A comment on Cookson, Drummond and Weatherly. (Response article). | Potential value of the methods for including equity within economic evaluations proposed by Cookson *et al.* | None provided. |
| Shiell (2009)^66^ | Still waiting for the great leap forward. (Response article) | Political issues associated with the inclusion of equity in economic evaluations. | None provided. |
| Weatherly *et al.* (2009)^58^ | Methods for assessing the cost-effectiveness of public health interventions: Key challenges and recommendations. (Full journal article) | Four key methodological challenges:  -Quantifying the effectiveness of interventions;  -Measuring and valuing outcomes;  -Inclusion of intersectoral costs and consequences;    -Inclusion of equity. | -More use could be made of techniques for analysing non-experimental data (eg. econometric analysis);  -Compensation test approach (Claxton *et al*.)  -Cost consequence analysis from perspective of each sector as additional analysis;  -None provided. |

| **Complex systems and multi-component interventions** | | | |
| --- | --- | --- | --- |
| **Author (year)** | **Title (type of article)** | **Key issues raised** | **Recommended approach** |
| Kelly *et al.* (2005)^59^ | Economic appraisal of public health interventions. (Briefing paper) | -QALY outcome may not be a sufficient measure;  -Greater need to develop methods for including equity considerations;  -It is important for a model to address what aspects of an intervention are successful/ unsuccessful. | -Cost consequence analysis from perspective of each sector as additional analysis; Discrete choice experiments may be used for valuation.  -None provided;  -None provided. |
| Plsek and Greenhalgh (2001)^68^ | Complexity Science: The challenge of complexity in health care. (Full journal article) | There is a challenge to address complexity within healthcare. | Point to the science of complex adaptive systems, but no specific approach described. |
| Rickles *et al*.(2009)^71^ | A simple guide to chaos and complexity. (Journal article ‘glossary’) | There are limitations associated with understanding causality, which is more complex in Public Health due to the risk factors (the determinants of health) often being social. | None provided. |
| Shiell *et al.* (2008)^76^ | Complex interventions or complex systems? Implications for health economic evaluation. (Full journal article) | -Public Health systems are complex, and as such they present new methodological challenges.  -The fact that Public Health interventions are often multi-component does not present new methodological challenges. | None provided. |
| Shiell and Hawe (1996)^69^ | Health promotion community development and the tyranny of individualism. (Full journal article) | Community impacts of interventions should be incorporated, which is more than the sum of the individual impacts. | None provided. |
| Smith and Petticrew (2010)^63^ | Public health evaluation in the twenty-first century: time to see the wood as well as the trees. (Full journal article) | -Public Health economic modelling should focus upon broader outcomes such as ‘happiness’;  -There is a need to focus on the direct and indirect effects of the interventions upon communities and populations, as well as on individual effects. | -None provided;  -None provided. |

| **Complex systems and multi-component interventions (cont)** | | | |
| --- | --- | --- | --- |
| **Author (year)** | **Title (type of article)** | **Key issues raised** | **Recommended approach** |
| Weatherly *et al.* (2009)^58^ | Methods for assessing the cost-effectiveness of public health interventions: Key challenges and recommendations. (Full journal article) | Four key methodological challenges:  -Quantifying the effectiveness of interventions;  -Measuring and valuing outcomes;  -Inclusion of intersectoral costs and consequences;    -Inclusion of equity. | -More use could be made of techniques for analysing non-experimental data (eg. econometric analysis);  -Compensation test approach (Claxton *et al*.)  -Cost consequence analysis from perspective of each sector as additional analysis;  -None provided. |
| Whitehead (2010)^70^ | The right wood, but barking up the wrong tree. (Commentary - response to Smith and Petticrew) | -There are Public Health interventions which have been undertaken using a macro-level analysis, contrary to what was discussed by Smith and Petticrew.  -It is the funders of Public Health economic modelling which encourage a micro-level approach rather than the analysts. | None provided. |
